# Dietary resilience among hunter-gatherers of Tierra del Fuego: Isotopic evidence in a diachronic perspective

**DOI:** 10.1371/journal.pone.0175594

**Published:** 2017-04-13

**Authors:** Mary Anne Tafuri, Atilio Francisco Javier Zangrando, Augusto Tessone, Sayuri Kochi, Jacopo Moggi Cecchi, Fabio Di Vincenzo, Antonio Profico, Giorgio Manzi

**Affiliations:** 1 Dipartimento di Biologia Ambientale, Sapienza Università di Roma, Rome, Italy; 2 CADIC – CONICET, Ushuaia, Argentina; 3 INGEIS-CONICET, Pabellón INGEIS, Buenos Aires, Argentina; 4 Dipartimento di Biologia, Università degli Studi di Firenze, Firenze, Italy; Max Planck Institute for the Science of Human History, GERMANY

## Abstract

The native groups of Patagonia have relied on a hunter-gatherer economy well after the first Europeans and North Americans reached this part of the world. The large exploitation of marine mammals (i.e., seals) by such allochthonous groups has had a strong impact on the local ecology in a way that might have forced the natives to adjust their subsistence strategies. Similarly, the introduction of new foods might have changed local diet. These are the premises of our isotopic-based analysis. There is a large set of paleonutritional investigations through isotopic analysis on Fuegians groups, however a systematic exploration of food practices across time in relation to possible pre- and post-contact changes is still lacking. In this paper we investigate dietary variation in hunter-gatherer groups of Tierra del Fuego in a diachronic perspective, through measuring the isotopic ratio of carbon (∂^13^C) and nitrogen (∂^15^N) in the bone collagen of human and a selection of terrestrial and marine animal samples. The data obtained reveal an unexpected isotopic uniformity across prehistoric and recent groups, with little variation in both carbon and nitrogen mean values, which we interpret as the possible evidence of resilience among these groups and persistence of subsistence strategies, allowing inferences on the dramatic contraction (and extinction) of Fuegian populations.

## Introduction

**Fig 1:**
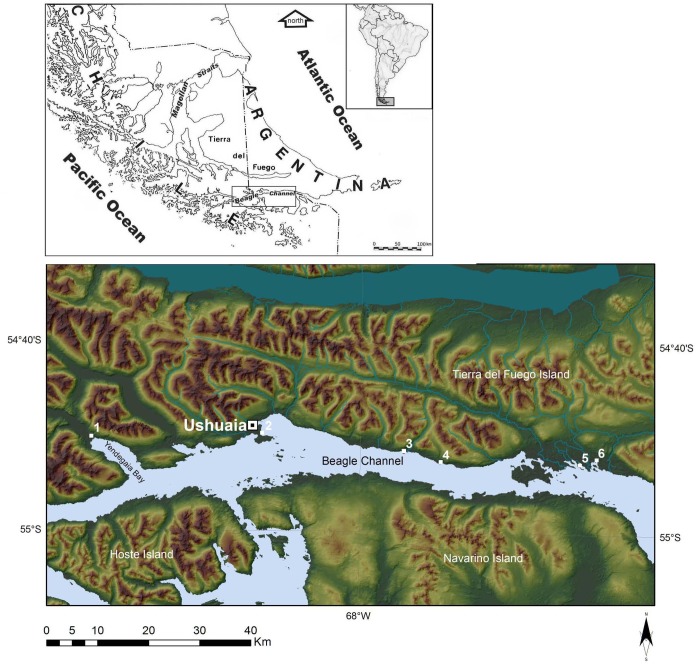
Map of the study area with location of the sites. Key: 1: Bove’s collection; 2: Aeroclub; 3: Shamakush-Mischiuen 3; 4: Paiashauaia 1; 5: Acatushun—Harberton Cementario; 6: Imiwaia.

The southern tip of South America is an archipelago of large and small islands that form an intricate network of channels. The landscape is characterized by mountainous terrain whose slopes are covered by a dense forest of the *Nothofagus* genus. The Beagle Channel is one of the main watercourses in this system, and runs from East to West almost in a straight line along the south coast of Isla Grande, separating it from the remaining islands of the south and southeast of the Tierra del Fuego archipelago (Fig 1).

This region was inhabited by maritime hunter-gatherers societies from 6400 BP until their nearly total transculturation in the late nineteenth century [1, 2]. These groups were generically referred to as *canoeros* (i.e., people of the canoes) and included Yámanas (or Yaghans) and Alacalufs. They inhabited two distinct areas in the Fuegian Archipelago in the late 19th century: the Yámanas populated the central and eastern portion of the Beagle Channel and the group of islands up to Cape Horn, while the Alacalufs occupied the western part of the Beagle Channel and of the Fuegian archipelago. The ethnographic sources available describe both groups as having a well-defined marine subsistence, with no significant differences in the dietary regime of these populations [3].

Earlier zooarchaeological studies on the Americas more in general and on Tierra del Fuego in particular have highlighted how the exploitation of aquatic resources, both marine and freshwater, was central to human adaptation in the area. They also pointed at the complexity of foraging strategies across the Late Pleistocene to historical times [4]. As an example, wide ranges of fish mobility were identified in the Holocene zooarchaeological record of the Beagle Channel and surrounding areas, where the exploitation of pelagic fish was also implied [5, 6].

Further, faunal analyses from archaeological sites across the Beagle Channel have highlighted the subsistence of these societies by the procurement of diverse marine and coastal animals, including marine mammals, guanacos, birds, fish, and mussels [1]. Although recent research has observed variations in human-animal relationships during the Middle-Late Holocene [5–7], pinnipeds have remained a critical dietary staple for these marine foragers. Moreover, some scholars believe that these resources were the basis of subsistence throughout the entire occupational sequence, providing an irreplaceable food source [2, 8]. In this regard, only in the nineteenth century, and with the arrival of European and North American sealers, the subsistence and behavioral patterns of these groups would have been seriously compromised [1, 2, 9, 10]. Hence, once contact was established, we should assume that the exploitation of the Patagonian landscape and the introduction of new foods by the Europeans have had an impact on the diet of the natives.

There is a generous literature on acculturation linked to colonialism, and its discussion goes beyond the scopes of this paper. We rather focus our analysis on some of the natural and cultural processes that might have ultimately contributed to the extinction of the Fuegians or their definitive assimilation by Western cultures [11]. By contrast, we consider several accounts on the cultural continuity and the ‘resistance’ (e.g. [12, 13]) of the native groups of Patagonia to external pressure, which appears to be linked to their foraging regime and a general cultural persistence throughout time.

One way to explore continuity vs. change in dietary practices after the arrival of European populations is by conducting an isotopic investigation on human remains in the region through a diachronic perspective. Earlier isotopic studies have explored diet among ancient groups in the Beagle Channel [14–17] and in the Western Archipelago [18, 19], confirming the evidence deriving from paleobotanical and faunal records. However very little is known about historical populations and variation throughout the contact period. One of the reasons for this was given by the paucity of human remains of Fuegian native populations of the 19th century.

The two skeletal collections of Fuegians recovered by Captain G. Bove in 1881 and 1883 and stored respectively in the Museo di Storia Naturale in Florence and the Museo di Antropologia G. Sergi of Sapienza University of Rome, in Italy offer new contribution to this discussion. The history of the recovery and the composition of the assemblages are fully described in a paper by Marangoni et al. [20]. Both assemblages are described in Bove’s reports [21], and were given to the Captain by Yaghans settled in the area of Yendegaia, near Ushuaia (Fig 1). Considering Bove’s notes, those human remains refer to family members to whom the natives related; we thus suggest a tentative attribution to the first half of the 19th century.

Pre-contact human bone remains in the research area were unearthed mainly through excavations conducted by the Proyecto Arqueológico Canal Beagle (Fig 1), and are currently preserved in the Fin del Mundo Museum, Ushuaia. Further bone remains corresponding to four individuals were donated to the Museo Etnográfico of Buenos Aires in the early twentieth century [17]. The information regarding mortuary contexts and excavation procedures has been described in Tessone [22], Piana et al. [23] and Vila et al [24]. In most cases human remains were found in primary position in shell middens and / or rock crevices. All samples are attributed to the late Holocene, among which only two individuals have been buried in historical phases: Acatushun and Harberton Cementerio [25].

Together with human remains, faunal samples gathered from different archaeological context were added to the dataset to provide an isotopic baseline. Most species selected are usually represented in the zooarchaeological record of the Beagle Channel as common food staples (with the exception of the fox).

## Materials and methods

We discuss here the isotope data of 42 humans and 166 animal specimens from different collections. The pre-contact subset excavated in the Beagle Channel counts 9 complete or almost complete skeletons (7 adults, 1 subadult and 1 infant). The post-contact subset excavated in the same region counts 2 almost complete skeletons (Acatushún and Harberton Cementerio), both corresponding to adults. Bone collagen extraction for stable isotopes analysis was carried out on ribs in good state of preservation. We included in the analysis data from comparative human material, such as the infant presented by Orquera and Piana [8], together with information reported by Yesner et al. [17] for the human remains of 4 adults from other areas of the archipelago.

The bone assemblages from Italy have a very different composition: the remains from Florence refer to a commingled set that arrived at the Museum in such a state, so that the skulls identified could not be reconnected with the post-cranial; on the basis of the crania observed we should consider a MNI of 12. Sampling for isotopic analysis was carried out on the skulls (namely, on portions of the vomer or the perpendicular plate of the ethmoid). The subset from Rome includes fifteen complete or almost complete skeletons (13 adults, 1 juvenile, 1 infant) in good state of preservation. Captain Bove recovered eleven of these during his trip, while the further samples were later acquired by the Rome museum through a donation. Sampling for isotopic analysis was carried out on the ribs of 14 individuals, with the exception of the infant, which was deliberately excluded to avoid damage to the skeletal elements (i.e., only the skull was preserved and sampling attempts resulted very invasive).

For all humans a general indication of sex and age at death of the individual is provided together with chronological attribution (Table 1).

**Table 1:** The human assemblage. Stable carbon and nitrogen isotope ratios for human collagen with data quality indicator (% of collagen %C, %N, C/N).

ID	Provenience	sample info	sex	age at death	cal. age (AD/BC)^a^	contact	% coll	∂^13^C _VPDB_	%C	∂^15^N _AIR_	%N	C/N
TF1	Rome	Fuegian1	f	40+	ca. 1800	post	28.2	-11.6	43.9	18.9	16.3	3.1
TF2	Rome	Fuegian2	f	20–30	ca. 1800	post	28.8	-11.9	45.6	18.9	17.1	3.1
TF3	Rome	Fuegian3	f	40+	ca. 1800	post	28.8	-11.2	46.0	19.3	17.2	3.1
TF4	Rome	Fuegian4	f	40+	ca. 1800	post	24.9	-11.9	42.8	18.4	15.9	3.1
TF5	Rome	Fuegian5	m	40+	ca. 1800	post	29.5	-12.0	44.8	18.3	16.5	3.2
TF6	Rome	Fuegian6	m	40+	ca. 1800	post	26.2	-11.9	46.6	18.0	17.2	3.1
TF7	Rome	Fuegiano7	f	40+	ca. 1800	post	26.6	-12.1	44.0	17.5	16.4	3.1
TF8	Rome	Fuegian8	m	30–40	ca. 1800	post	18.7	-12.1	44.3	17.6	16.6	3.1
TF9	Rome	Fuegian9	f	40+	ca. 1800	post	26.0	-12.5	43.8	17.5	16.4	3.1
TF10	Rome	Fuegian10	f	20–30	ca. 1800	post	29.3	-11.2	45.4	18.0	16.9	3.1
TF11	Rome	Fuegian11	f	40+	ca. 1800	post	39.8	-11.3	41.4	18.6	15.4	3.1
TF12	Rome	Fuegian12	m	15	ca. 1800	post	29.4	-11.6	46.6	17.4	17.4	3.1
TF13	Rome	Fuegian13	f	16	ca. 1800	post	26.2	-11.6	43.6	17.6	16.2	3.1
TF14	Rome	Fuegian13A	m	40+	ca. 1800	post	24.4	-13.0	43.2	17.5	15.8	3.2
TF15	Florence	3116	f	adult	ca. 1800	post	25.1	-12.5	43.4	19.4	15.4	3.3
TF16	Florence	3119	f	adult	ca. 1800	post	23.5	-12.3	43.4	18.6	15.8	3.2
TF17	Florence	3120	f	adult	ca. 1800	post	27.4	-12.1	40.1	19.0	14.7	3.2
TF18	Florence	3122	m	adult	ca. 1800	post	23.1	-12.3	38.9	19.7	14.3	3.2
TF19	Florence	3124	m	adult	ca. 1800	post	20.9	-12.0	42.4	19.3	15.5	3.2
TF20	Florence	3126	m	adult	ca. 1800	post	24.1	-11.4	45.6	19.6	16.2	3.3
TF21	Florence	3127	m	adult	ca. 1800	post	24.5	-12.5	42.0	19.6	15.1	3.2
TF22	Florence	3129	m	adult	ca. 1800	post	22.4	-12.1	45.3	20.4	16.5	3.2
TF23	Florence	3131	m	adult	ca. 1800	post	24.9	-12.0	43.3	19.7	15.9	3.2
TF24	Florence	3133	m	adult	ca. 1800	post	17.5	-12.1	45.8	19.5	16.8	3.2
TF25	Florence	3132	f?	adult	ca. 1800	post	25.6	-12.5	43.2	18.7	15.7	3.2
TF26	Florence	3134	m	adult	ca. 1800	post	18.5	-15.0	45.1	12.3	16.3	3.2
TF 27	MFM	Mischiuen III	f	13–17	1300–1400	pre	n.d.	-11.1	46.5	18.1	16.6	3.3
TF 28	MFM	2668	n.d.	adult	n.d.^d^	pre	n.d.	-11.5	43.1	18.2	15.4	3.3
TF 29	MFM	2669	n.d.	adult	n.d.^d^	pre	n.d.	-13.3	41.5	15.8	15.1	3.2
TF 30	MFM	795	n.d.	adult	n.d.^d^	pre	n.d.	-11.9	45.0	18.0	16.0	3.3
TF 31	MFM	1607	n.d.	juvenile	n.d.^d^	pre	n.d.	-11.9	40.3	18.4	14.4	3.3
TF 32	MFM	SHE	m	35–45	520–650	pre	n.d.	-12.4	41.4	18.3	14.7	3.3
TF 33	MFM	Acatushun	f	30–40	1600–1800	post	n.d.	-13.9	46.4	16.7	16.5	3.3
TF 34	MFM	Paiashauaia	f	35–45	530–670	pre	n.d.	-11.9	43.9	18.3	15.7	3.3
TF 35	MFM	Aeroclub	n.d.	adult	pre-contact^e^	pre	n.d.	-11.2	40.6	19.2	14.8	3.2
TF 36	MFM	BI1	f	25–49	1300–1415	pre	n.d.	-11.2	42.3	19.2	15.4	3.2
TF 37	MFM	Harberton	m	25–35	1600–1800	post	n.d.	-11.6	42.6	18.6	15.4	3.2
TF 38^b^	M. etngráfico	Isla Hoste	n.d.	adult	pre-contact^f^	pre	n.d.	-13.3	n.d.	17.2	n.d.	n.d.
TF 39^b^	M. etngráfico	Ushuaia	n.d.	adult	pre-contact^f^	pre	n.d.	-12.6	n.d.	18.8	n.d.	n.d.
TF 40^b^	M. etngráfico	Isla Hoste	m	adult	pre-contact^f^	pre	n.d.	-16.8	n.d.	13.2	n.d.	n.d.
TF 41^b^	M. etngráfico	Isla Navarino	m	adult	pre-contact^f^	pre	n.d.	-18.5	n.d.	10.6	n.d.	n.d.
TF 42^c^	---	Shamakush I	n.d.	0–6 months	950–1300	pre	n.d.	-12.8	n.d.	---	n.d.	n.d.

Key: f = female, m = male; n.d. = not determined.

^a^ Radiocarbon data was calibrated using SHCal04 curve from Calib Rev 6.0.1 program

^b^ published in Yesner et al., 2003

^c^ published in Orquera and Piana, 1996

^d^ Undated sample from unknown depositional context

^e^ prehistoric midden

^f^ date within the last 1500 years before contact

The faunal bones were recovered from archaeological sites on the north coast of the Beagle Channel, spanning from the Middle to the Late Holocene (Table 2), published in detail in Zangrando et al. [26, 27] and Kochi [28]. They are not directly associated with the Argentinian subset, given that most sites excavated were primary burials outside of residence areas and did not provide a faunal record. They are however comparable with the human remains in term of location and chronological attribution. In detail, the subset of terrestrial animals is composed of 32 samples of guanacos (*Lama guanicoe*) and 3 samples of andean fox (*Lycalopex culpaeus*). The aquatic group is more diverse: it comprises 46 samples of southern fur seals (*Arctocephalus australis*), 2 samples of otter (*Lontra provocax*), 10 samples of albatross (*Thalassarche* sp.) and 11 cormorants (*Phalacrocorax* sp.). It further includes 45 samples of pelagic fishes, which include hakes (*Macronus magellanicus*), snoeks (*Thyrsites atun*), southern hakes (*Merluccius* sp.), together with 2 samples of coastal fish species (*Eleginops maclovinus*). We further discuss data on modern samples of mussels (N = 15), partially presented in Zangrando et al. [26, 27] and Kochi [28].

**Table 2:** The faunal assemblage. Mean carbon and nitrogen ratios and C/N (with standard deviation) for animal taxa examined.

Taxa	common name	N	∂^13^C _VPDB_		∂^15^N _AIR_		C/N	
Terrestrial			mean	sd	mean	sd	mean	sd
*Lama guanicoe*	guanacos	32	-20.6	2.2	0.5	2.5	3.2	0.03
*Lycalopex culpaeus*	andean fox	3	-18.3	2.3	9	3.3	3.3	0.06
**Riverine-marine**								
*Lontra provocax*	otter	2	-10.7	1.8	21.1	4.2	3.2	0
*Eleginops maclovinus*	Patagonian blenny	2	-12.1	0.5	16.4	0.1	3.6	0.2
**Marine**								
*Arctocephalus australis*	seal	46	-11.8	0.5	17.3	0.6	3.2	0.04
*Thalassarche sp*.	albatross	10	-13.1	0.5	18.6	0.9	3.1	0.02
*Phalacrocorax sp*.	cormorants	11	-12.7	0.3	15.4	0.6	3.2	0.05
*Macruronus magellanicus*	hakes	14	-11.6	0.3	15.8	0.9	3.2	0.02
*Thyrsites atun*	snoeks	14	-12.6	0.7	15.3	0.9	3.2	0.04
*Mytilidae*	mussels	15	-16.2	0.6	11.1	0.5	3.2	0.05
*Merluccius sp*.	hake	17	-12.8	0.7	17	0.9	3.3	0.05

Collagen extraction and sample preparation for isotopic analysis followed two slightly different methods, given that analyses were run at respective laboratories in Italy and Argentina. Both methods derive from Longin’s [29] standard, as described below.

### Italian subset

Following Brown et al. method [30], cortical bone (0.5 g) was cleaned by sand abrasion and demineralized in 0.5M solution of HCl at 4°C for at least four days. The samples were then rinsed to neutral pH and gelatinized in pH 3 HCl at 70°C for 48 hours. The collagen solution was filtered off with 5–8 μm Ezee filters, frozen, and then freeze-dried. Each of the collagen extracts was weighed (ca. 1 mg) in triplicate into tin capsules, and stable carbon and nitrogen isotope ratios were measured using an automated elemental analyzer coupled in continuous-flow mode to an isotope-ratio-monitoring mass spectrometer (Costech elemental analyzer coupled to a Thermo Finnigan MAT253 mass spectrometer). Analysis was carried out at the Godwin Laboratory, University of Cambridge (UK).

### Argentinian subset

Bone fragments were cleaned with abrasive elements and ultrasonic baths. Each sample of approximately 0.3g was soaked in dilute HCl (0.5%), which was changed every 24–48 h. Then, it was rinsed with distilled water and treated with 0.125% solution of NaOH for 20 hrs. Finally, the collagen extracted was dried in an oven at 40°C for hrs. [31].

Measurement of ^13^C/^12^C and ^15^N/^14^N ratios in the collagen fraction was performed with a Carlo Erba EA1108 Elemental Analyzer (CHN), connected to a continuous flow Thermo Scientific Delta V Advantage mass spectrometer through a Thermo Scientific ConFlo IV interface.

For both subsets stable isotope results are expressed as the ratio of the heavier isotope to the lighter isotope (^13^C/^12^C or ^15^N/^14^N) and reported as ∂ values in parts per thousand (‰), relative to internationally defined standards for carbon (Vienna Pee Dee Belemnite, VPDB) and nitrogen (Ambient Inhalable Reservoir, AIR). Based on replicate analyses of international and laboratory (i.e., alanine, nylon, caffeine, USGS40, EMC) standards, measurement errors are less than ± 0.2‰ for ∂^13^C and ∂^15^N. The collagen yield, the percentage of carbon and nitrogen, and the atomic C/N ratio of each sample were also recorded to check collagen quality [32–34]. Finally, the modern fauna samples were corrected for carbon by adding 1‰ due to the Suess effect [35]. The historic human samples of Bove’s collections were not corrected for this effect [36], considering their estimated chronology.

Results obtained where analyzed through descriptive statistics and non-parametric tests using IBM SPSS ver. 23.

## Results

Table 1 provides data on collagen yields, C/N ratios and collagen isotopic values of the sampled humans, with mean carbon and nitrogen isotope values and C/N ratios from collagen of animals presented in Table 2. For all the samples studied collagen yield and C/N ratios confirm that isotopic signals are to be considered reliable.

Carbon isotope values of collagen from terrestrial animals are typical of mammals feeding on C_3_ plants (mean ∂^13^C between -18.3‰ and -20.6‰). Variation in nitrogen isotope values (∂^15^N) is highest, and it ranges from 9‰ (fox) to 0.5‰ (guanaco); the very low values for the guanacos can be explained by some consumption of lichens (*Usnea* sp.) with very low ∂^15^N values (-10.8 to -17.5 ‰) [28].

As expected, the marine vertebrate resources have higher ∂^13^C and ∂^15^N values than the terrestrial herbivores, with means of -12.2‰±0.8‰ and 17.1‰±1.9‰ respectively. Little variation in isotope means is observed for seals, seabirds and fish. Three species of pelagic fish (*Macruronus magellanicus*, *Thyrsites atun*, *Merluccius* sp.) have ∂^13^C values with means ranging from -11.6‰ to -12.8‰, and mean ∂^15^N values range from 17.0‰ to 15.3‰. Carbon isotope values from collagen of seabirds and southern fur seal are similar to those for pelagic fish. For ∂^15^N values, it is worth noticing that albatrosses have higher mean values than other marine resources. In the other extreme of the marine food web, mussels are presented with the lower mean values for ∂^13^C (-16.2‰±0.6) and ∂^15^N (11.1‰±0.5), which are significantly different from those of the marine vertebrates.

Among the estuary resources, *Lontra provocax* has much heavier isotope mean values than marine resources (-10.7‰ for ∂^13^C and 21.1‰ for ∂^15^N), and *Eleginops maclovinus* has analogous values to those of pelagic fish.

**Fig 2:**
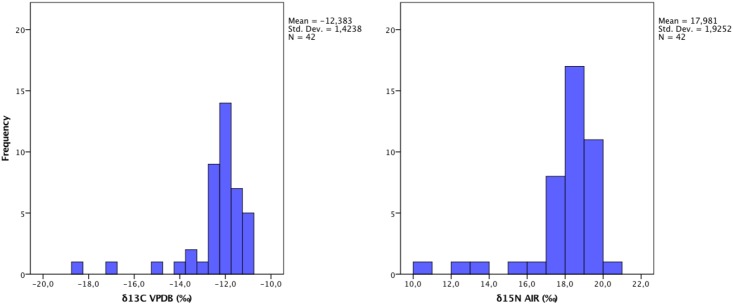
Distribution of the human data. Frequency of stable carbon and nitrogen isotope values.

The ∂^13^C and ∂^15^N values of human remains range from almost completely marine-derived diets (-11.1‰ and 18.1‰) to values that could reflect entirely terrestrial food composition (-18.5‰ and 10.6‰). However, more than 90% of values are grouped in the ranges -11.1 and -13.3 ‰ for ∂^13^C and 18.1 and 17.2 ‰ for ∂^15^N, which indicates mainly marine-based diets (Fig 2).

**Fig 3:**
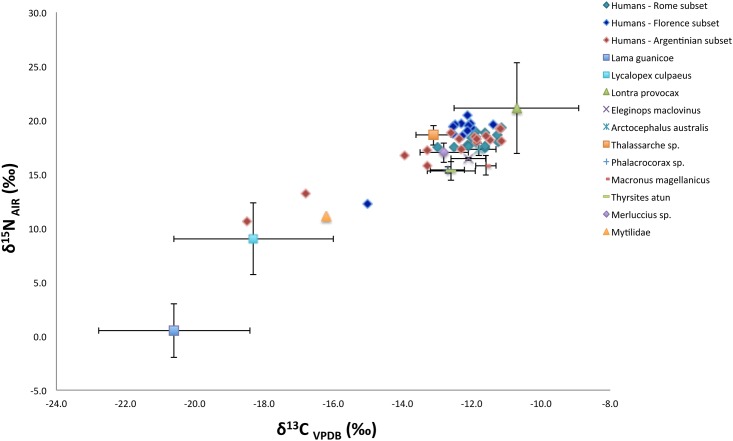
Stable carbon and nitrogen isotopes biplot. ∂^13^C and ∂^15^N (‰) of human samples with associated animal mean values and standard deviation. The three human subsets are kept separate.

The mean ∂^13^C and ∂^15^N values of the humans are -12.4±1.4‰ and 18.0±1.9‰, and the medians respectively -12.1‰ and 18.4‰. If we exclude the few cases (N = 3) that could reflect up to 50% terrestrial food composition, the means and medians do not significantly change: mean ∂^13^C and ∂^15^N values are -12.0±0.6‰ and 18.5±0.9‰, and the medians respectively are -12.0‰ and 18.5‰. The correlation between ∂^13^C and ∂^15^N in human values is significant (r^2^ = 73.9 p = 0.0001) (Fig 3).

In general, the humans cluster in a rather homogeneous group with no significant differences between males and females measured in either carbon or nitrogen values for the three subsets (S1 Table).

## Discussion

The transculturation from nomadic/mobile hunting-gathering to sedentary/residential lifestyle is a key process in the history of the Fuegian populations. The overexploitation of pinnipeds produced by European and American sealers, the demographic concentrations in missions and ranches, and the introduction of illnesses by Europeans during the 19^th^ century have produced deep structural changes in the subsistence patterns and demography of native groups. Our data and statistical analysis suggest no differences in dietary practices across time detectable at an isotopic level, which is in counter-tendency with our expectations, and offer some interesting convolution.

**Fig 4:**
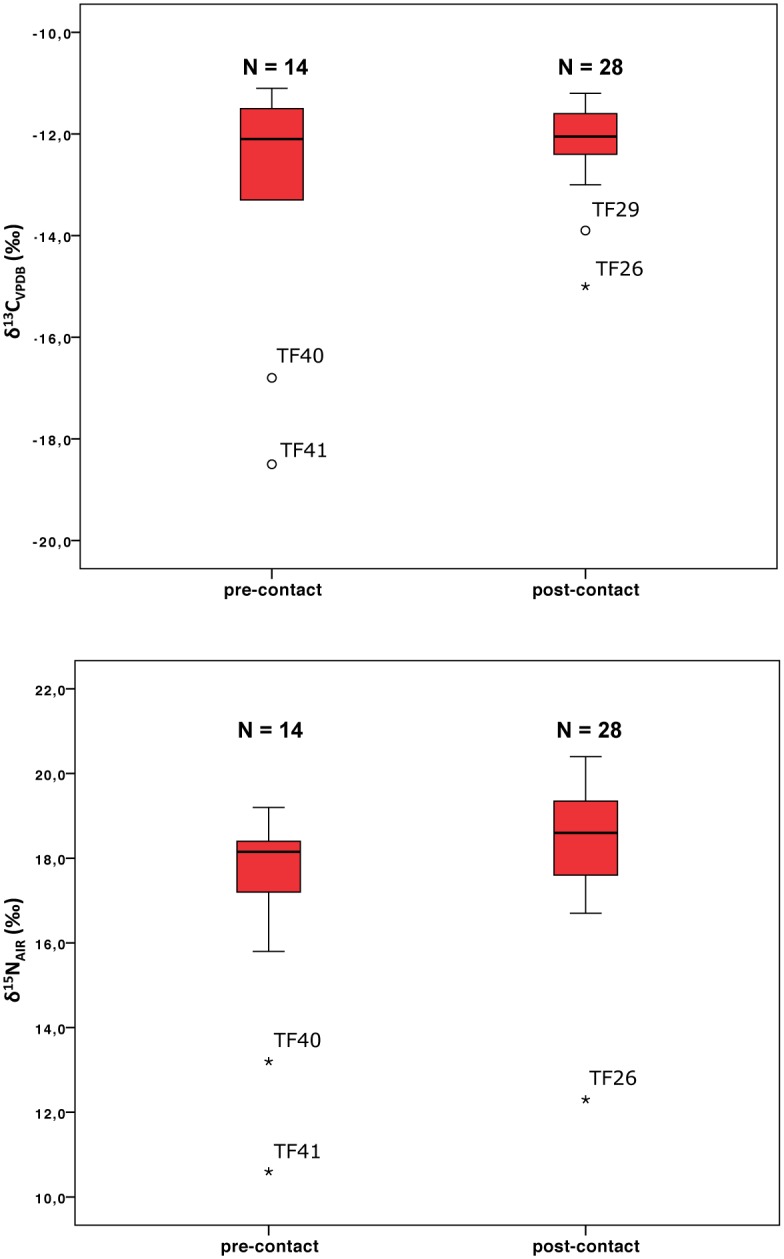
Pre- and post-contact data distribution. Box and whisker plot of nitrogen and carbon values for pre- and post-contact human populations. Nitrogen median values show little differences between pre-contact and post-contact phases.

The Kolmogorov-Smirnov test shows that there is no statistical difference in ∂^15^N between pre- and post-contact populations (p = 0.185; n = 14 and n = 28 respectively), with slight differences in the distribution of data (S1 Fig). For nitrogen values, isotope data show that post-contact populations kept diet focused on marine resources of high trophic levels. This is particularly interesting: if seals—one of the preferred food sources among the Yamana—were becoming less available because of Europeans and Americans’ exploitation we should assume post-contact consumption of low trophic level species and higher values among pre-contact groups, as a consequence of consumption of high trophic level species. Surprisingly, the post-contact nitrogen data cluster around higher values, against this expectation (Fig 4). However, the reported nitrogen values for some species of seabirds and pelagic fish are similar, and even higher, to those of fur seals in the southern South Atlantic, indicating analogous trophic levels. Therefore, a change in subsistence redirected towards the exploitation of such minor resources should not necessarily have implied a change in the trophic levels.

The descriptive statistics presented in Table 3 is also noteworthy: it shows a difference of 1.2‰ between the mean ∂^15^N values, and a lesser distance between the medians (0.5‰), which do not support a significant change in trophic terms.

**Table 3:** Summary of pre- and post-contact data. Descriptive statistics for the human sample only, according to chronological phase.

	∂^13^C _VPDB_		∂^15^N _AIR_	
	Pre-contact	Post-contact	Pre-contact	Post-contact
N	14	28	14	28
Min.	-18.5	-15.0	10.6	12.3
Max.	-11.1	-11.2	19.2	20.4
Mean	-12.8	-12.2	17.2	18.4
Stand. Dev.	2.1	0.8	2.5	1.5
Median	-12.3	-12.1	18.2	18.7

We prompt to think that this difference in the mean ∂^15^N values could be describing a variation in the diet, indicating the consumption of fewer lower marine trophic level resources or more high-trophic level marine species. A decrease in the consumption of shellfish by post-contact populations might be supported by historical documents: the setting-up of the Anglican Mission in Ushuaia by the end of 1860s led to the demographic concentration and semi-sedentism of the native population in few years. There were almost permanently residing between 10 and 18 Yamana families, and every year the Mission received hundreds of Yamana people where they stayed from four to six months [37–39]. Such concentration of persons and such reduction in mobility certainly should have resulted in the overexploitation of local resources, especially shellfish [1]. Given the ∂^15^N lighter values on shellfish in comparison with other marine resources, the high nitrogen values in post-contact human remains could be describing accordingly less accessibility to those resources.

The Fuegian landscape shows little seasonal variation in the environment, with oxygen isotopic analyses indicating a shift in mean annual temperature that ranges between 1 and 2°C across several centuries (i.e., between the Medieval Climatic Anomaly and the Little Ice Age) [40], with paleobotanical data [41, 42] suggesting little changes in the local vegetation over the last six millennia. Similarly, archaeological data [43] show that human occupation in the area is uninterrupted, with a general uniformity in cultural practices. Across time human groups settled in Tierra del Fuego display a general continuity, either in cultural practices or in technological complexity, with rare innovations. This appears to be true also for what concerns diet, as types of food consumed and levels of protein intake appear to remain unchanged across several centuries. According to Orquera and colleagues [43], “in spite of its simplicity, the system was successful. Its collapse happened by the end of the 19th century, and was caused by the catastrophic pinniped depredation carried out by Euro-American, Chilean and Argentine groups, as well as by the introduction of sickness against which the indigenous populations had no immunity”. Under such circumstances, it is possible that the subsistence patterns of the marine hunter-gatherers of the Beagle Channel based on the exploitation of a high diversity of resources have given to these populations a resilience to cope with the hunting of fur seals by industrial populations. The overall consistency in isotope data might hence be interpreted in cultural terms. We propose here that: i) post-contact societies might have experienced a significant reduction in the consumption of pinnipeds, which was compensated through the intake of other aquatic mammals (i.e., seabirds and fish with similar nitrogen levels), with effects of such shift in the diet not detectable at the isotopic level; at the same time, ii) a significant decrease in the consumption of molluscs by post-contact populations might have driven up their nitrogen values;

The human ecological resilience of the Fuegian groups expressed in the search for nutrional alternatives can be perceived, as anticipated, as the evidence of a cultural resistance, which goes along with a general stability of these people. Here, the idea of resistance is not linked to noncompliance [44], as in the lack of negotiation of traditions. Rather, it is perceived as the tendency to persist in the expression of specific traditions (the hunting/fishing of aquatic species), despite external stimuli and/or the knowledge of alternatives. For the Fuegians, such resistance seems to translate in a striking uniformity of dietary practices across several centuries, as mirrored in the isotopic ratios of the skeletal tissues. Furthermore, considering that the contribution to collagen of different types of dietary proteins appears to remain constant throughout time, we might need to reconsider the influence of diseases as the main factor in the dramatic contraction (and eventually extinction) of Fuegian populations.

## Supporting information

S1 Table Statistics report. Summary of the Mann-Whitney U test for human carbon and nitrogen data according to sex. The three subsets are kept separate. For the Ushuaia subset 7 individuals were excluded, as no sex estimate was available.
(DOCX)

S1 Fig Biplot of stable carbon and nitrogen data. Mean humans∂^13^C and ∂^15^N values (with sd) for pre-contact (n = 14) and post-contact (n = 28) subsets.
(DOCX)
