# Supplementary material for: Dietary resilience among hunter-gatherers of Tierra del Fuego: Isotopic evidence in a diachronic perspective
Source: PLoS One. 2017 Apr 13;12(4):e0175594. doi: 10.1371/journal.pone.0175594 (PMC5391079; doi:10.1371/journal.pone.0175594)
Supplement: S1 Table — Summary of the Mann-Whitney U test for human carbon and nitrogen data according to sex. The three subsets are kept separate. For the Ushuaia subset 7 individuals were excluded, as no sex estimate was available. (DOCX) [file pone.0175594.s001.docx]

**S1 Table. Statistics report**. Summary of the Mann-Whitney U test for human carbon and nitrogen data according to sex. The three subsets are kept separate. For the Ushuaia subset 7 individuals were excluded, as no sex estimate was available.

| **Subset** | **N** | **p value**  **δ^13^C _VPDB_** | **p value δ^15^N _AIR_** |
| --- | --- | --- | --- |
| Florence | 12 | 0.283 | 0.073 |
| Rome | 14 | 0.147 | 0.147 |
| Argentina | 9 | 0.190 | 0.556 |
