# Supplementary material for: Dietary resilience among hunter-gatherers of Tierra del Fuego: Isotopic evidence in a diachronic perspective
Source: PLoS One. 2017 Apr 13;12(4):e0175594. doi: 10.1371/journal.pone.0175594 (PMC5391079; doi:10.1371/journal.pone.0175594)

**S1 Figure. Biplot of stable carbon and nitrogen data.** Mean humans ∂^13^C and ∂^15^N values (with sd) for pre-contact (n=14) and post-contact (n=28) subsets.


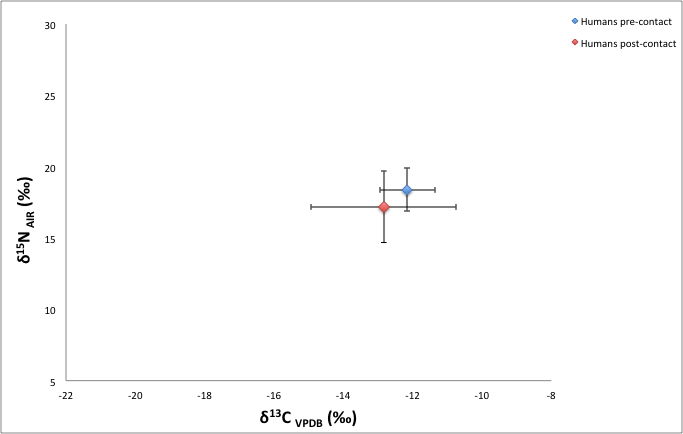

Supplement: S1 Fig — Mean humans∂13C and ∂15N values (with sd) for pre-contact (n = 14) and post-contact (n = 28) subsets. (DOCX) [file pone.0175594.s002.docx]
